# Clinical Features and Physical Properties of Gummetal Orthodontic Wire in Comparison with Dissimilar Archwires: A Critical Review

**DOI:** 10.1155/2021/6611979

**Published:** 2021-01-28

**Authors:** Krzysztof Schmeidl, Joanna Janiszewska-Olszowska, Katarzyna Grocholewicz

**Affiliations:** Department of Interdisciplinary Dentistry Pomeranian Medical University in Szczecin, Poland

## Abstract

**Objective:**

Gummetal is a novel multifunctional alloy which possesses distinctive properties with the potential to refine and amend the efficacy of orthodontic treatment. The objective of this critical literature review was to investigate scientific evidence concerning the mechanical and clinical features of this recently manufactured beta-titanium orthodontic wire.

**Materials and Methods:**

Electronic databases: PubMed, PMC, Google Scholar, Ovid, and Cochrane Library were searched. Studies investigating the properties of Gummetal orthodontic wire including in vitro and clinical studies were selected, validity was assessed, and data was extracted. The risk of bias was assessed by the Cochrane risk of bias Tool 2.0 in a randomized clinical trial. *Results and Discussion*. Among 322 papers, 13 papers were selected and divided into two groups: prospective double-blinded randomized clinical trial and in vitro studies.

**Conclusions:**

The results of this review should be interpreted with caution because of the heterogeneity of the studies. Only single clinical trial paper was found in the literature. The studies reported different characteristics obtained by various methods; thus, it was difficult to objectively compare the results. Low bending strength, low fatigue limit, and high resilience have been confirmed. Gummetal provides lower force than Nitinol and TMA but higher than Supercable wire. Plastic deformation of Gummetal questions its superelasticity. Friction of Gummetal wire is comparable to SS and CoCr wires. Because of its nontoxic chemical composition, Gummetal might be useful in the initial phase of orthodontic treatment for patients suffering from nickel allergy. Further studies are necessary to assess the usefulness of Gummetal in the clinical practice.

## 1. Background

Orthodontists use archwires in multibracket appliances in order to achieve a 3D control of tooth movement in the active phase of orthodontic treatment. The force moving the teeth is provided by the elasticity of orthodontic wire. In the Angle and Tweed era, only gold-nickel alloy wire resisted corrosion and was elastic enough to make it available for orthodontic treatment. In 1933, in the United States, Rocky Mountain Orthodontics started producing cobalt-chromium (CoCr) (Elgiloy). It had similar strength and Young Modulus as gold-nickel wire but was much cheaper and quickly became the material of choice. For years, CoCr and stainless steel (SS) wires have been a standard in orthodontic treatment [1].

In the 1970s, nickel-titanium (NiTi) wire started the next era in orthodontics. Its superelasticity and shape memory simplified the initial phase of orthodontic treatment. However, it is also almost impossible to bend which makes it inadequate for the middle and final phases of orthodontic treatment [2]. In addition, NiTi alloy contains 50% of nickel, which may provoke the body to produce antibodies in allergic reaction [3]. Elimination of heavy metals from orthodontic wires was desired.

Beta-titanium (*β*-Ti) alloys are the next and a very important class of nickel and chromium-free alloys that have found use in demanding medical applications such as orthodontic and orthopedic implants and orthodontic wires. For orthodontic use, bendable titanium-molybdenum (TiMo) wires were fabricated with similar properties to CoCr and NiTi alloys [4]. *β*-Ti wires are suitable to replace CoCr and SS wires, but, unfortunately, they are not good for applications in which NiTi alloys flexibility is required.

The search for an ideal orthodontic wire has not come to an end yet. Metals and alloys designed for biomedical applications require specific properties, such as an exceptional biocompatibility, lack of toxicity, and good corrosion resistance [5]. The perfect orthodontic archwire should be aesthetic, very elastic, formable, and easy to bend with high tensile strength as well. Moreover, it should provide low coefficient of friction moving the teeth efficiently and be able to control the orthodontic force freely.

Gummetal is a novel multifunctional *β*-Ti alloy developed in Japan in 2001 at the Metallurgy Research Section of Toyota Central R&D, Inc. It consists of titanium, niobium, tantalum, zirconium, and oxygen. Gummetal's chemical composition (Ti-23Nb-0.7Ta-2Zr-1.2O) was based on its atomic valence theory [6]. This alloy is intensively cold-worked to produce its important characteristics. Gummetal properties arise from the juxtaposition of three electronic magical numbers: a compositional average valence electron number of 4.24 valence electrons per atom, bond order (BO-value) of 2.87, and Md value of 2.45 eV (“d” electron-orbital energy level). According to the producers, this nontoxic alloy combines not only high strength but also very high elasticity due to the extremely low value of Young's modulus, which is exceptionally rare for a metal alloy to possess both of these properties at the same time. In this novel alloy, as stated by the manufacturers, plastic deformation (the dislocation motion of the crystal) is controlled completely which makes it unique [7].

As claimed by Hasegawa [8], Gummetal appears to be almost ideal material for orthodontic archwire. It is assumed to produce a small continuous force from an early stage of crowding treatment. Because Gummetal does not follow Hooke's law, it could lessen the orthodontic force even with large teeth displacement.

The aim of the present study was to verify and confirm the advertised properties of this novel *β*-Ti archwire as extremely low elastic modulus, super high tensile strength, high flexibility, nontoxicity, dislocation-free plastic deformation mechanism, low coefficient of friction, low bending strength, and low fatigue limit.

## 2. Materials and Methods

Literature search was proceeded in February 2020 using the keywords: “gummetal orthodontic wire” in the following databases:

- PubMed
- PMC
- Google Scholar
- Ovid
- Cochrane Library

**Figure 1:**
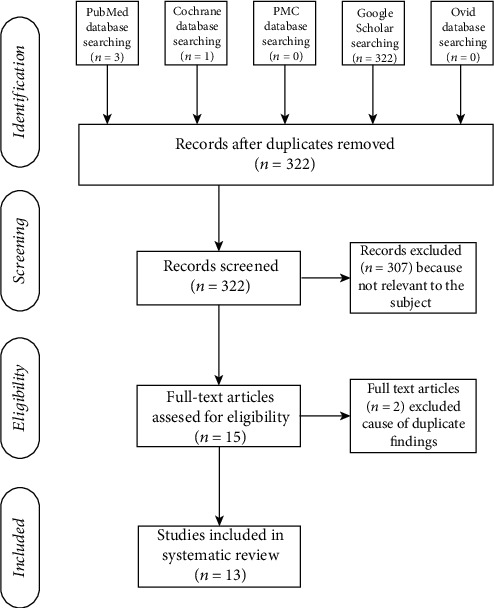
PRISMA flow diagram.

The PRISMA flow diagram is presented in Figure 1.

All titles and abstracts were read by the first and verified by the second author to retrieve eligible studies. Then, full texts of papers included were obtained. No date or language limits were applied if at least article's abstracts were in English. The inclusion criteria comprised (1) double-blinded randomized clinical trials (RCT), (2) controlled clinical trials, and (3) in vitro studies. The exclusion criteria were (1) papers not investigating directly the properties of Gummetal orthodontic wire, (2) reviews, (3) authors' debates, (4) abstracts, (5) editorials, (6) opinions, and (7) case reports. All full texts of papers included were retrieved and analyzed. Hand search was proceeded in reference lists of the studies included. The authors used Cochrane risk of bias (RoB) Tool 2.0 to assess the RoB of the selected RCT [9].

## 3. Results

Initially, fifteen papers proved eligible for a critical review. However, two of them had to be excluded because of the duplication of the findings described. Two studies (one article and one dissertation) covered the same double-blind RCT and were cowritten by the same first author. Another group of authors published the same findings twice: in Japanese (2013) and in English (2015). Surprisingly, there was only one RCT paper found in the literature (Table 1).

All the latter were in vitro studies (Table 2).

**Figure 2:**
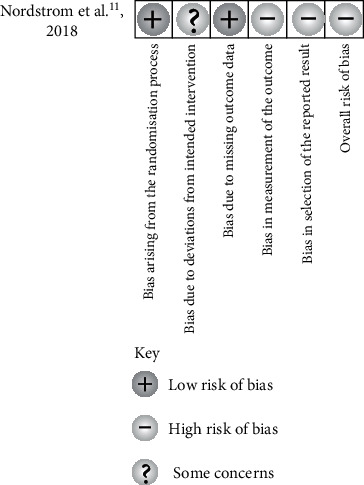
Cochrane risk of bias tool 2.0 results.

In included RCT study, the authors had some concerns regarding the bias due to deviations from intended intervention. The clinicians were aware of participants' assigned group during the trial. However, the patients were not most likely conscious of archwire used and that presumably have not affected the outcome of the intervention. Two domains were judged to present high RoB. The overall RoB of this study was evaluated high which corresponds to the worst risk of bias in any of the domains (Figure 2) [9].

### 3.1. Microstructure and Mechanical Properties

The microstructure and mechanical properties of cold-drawn and annealed TNTZO (Ti-Nb-Ta-Zr-O, Gummetal) wires (prepared by powder metallurgy) with 0.3 mm diameter were analyzed by Zhang et al. [11]. The microstructure of cold drawn TNTZO consisted of nanometer-sized elongated drains “marble-like” in cross-section with 70 nm width. After 700°C 5 min annealing, the grain size increased to approximately 5 *μ*m. The cold-drawn wires exhibited better mechanical properties, higher tensile strength (around 1000 MPa) and similar Young's Modulus (69 MPa) compared to annealed wires. In addition, TNTZO wire presented higher creep resistance and lower stress exponent compared to titanium (Ti) and TC4 wires of the same diameter.

### 3.2. Initial Teeth Alignment and Force System Evaluation

The clinical efficiency of 0.016^″^ Gummetal and 0.016^″^ NiTi orthodontic wires during teeth alignment in the first two months of treatment was compared by Nordstrom et al. [10] in a prospective, double-blind randomized clinical trial. Twenty-eight patients were divided into two equal groups. During the treatment, digital scans were performed and then used to assess changes in Little's Irregularity Index and the alteration in intercanine and intermolar widths. With Gummetal wire, there was 27% crowding reduction during the first month, and an additional 25% decrease in crowding was observed in the following month. There was no significant difference observed in the decrease in irregularity between the two groups over time. Moreover, there was no significant difference between the groups concerning the changes in intercanine and intermolar width.

The investigation of the initial force systems of Gummetal and its comparison to the Supercable, Nitinol and TMA archwires proved that Gummetal provided slightly lower (10% lower) force systems than Nitinol and higher than Supercable, which was the only archwire that has not exceeded the recommended values [12]. TMA delivered the highest force value. Moreover, the author noticed a plastic deformation after removing the archwires from the brackets in 7% of Nitinol wires, 60% of Gummetal wires, and 83% of TMA wires.

### 3.3. Bending Properties, Fatigue Evaluation

Thirteen different 0.016^″^ × 0.022^″^*β*-Ti archwires (including Gummetal), SS, and NiTi wires were tested by Suzuki et al. [13] for stiffness, active deflection range, load at 3 mm displacement, and apparent plastic deformation. Among the wires tested, Gummetal presented the lowest stiffness (below 3 N/mm), second highest active deflection range after NiTi (approximately 1.75 mm), second lowest load at 3 mm deflection after NiTi (approximately 6 N), and apparent plastic deformation.

High-cycle fatigue behavior in three *β*-Ti wires (TMA 0.016^″^ × 0.022^″^, Resolve 0.016^″^ × 0.022^″^ and Gummetal 0.017^″^ × 0.022^″^) was analyzed by Murakami et al. [14] using static bending test and bending fatigue test. Among all wires studied, Gummetal exhibited the lowest elastic modulus, fatigue limit, and bending strength. It also performed the highest resilience. However, there was no difference observed in the number of cycles to failure among these three archwires. TMA, Gummetal, and Resolve presented similar risk of the archwire fracture.

### 3.4. Frictional Force (FF)

The frictional forces (FFs) of titanium-niobium (TiNb, Gummetal), NiTi, and TiMo archwires in sizes 0.016^″^, 0.016^″^ × 0.022^″^, and 0.017^″^ × 0.025^″^ (in 0.018^″^-slot bracket) and 0.018^″^, 0.017^″^ × 0.025^″^, and 0.019^″^ × 0.025^″^ (in 0.022^″^-slot bracket) ligated with elastic modules at three different wire-bracket angles were compared by Takada et al. [15]. It has been revealed that the FFs increased gradually with the angle and size of the wire in both types of brackets. Moreover, Gummetal and NiTi archwires exerted comparable FFs, and those of TiMo presented the greatest FFs. In this study, all three alloys generated greater FFs in the 0.018^″^-slot bracket than in the 0.022^″^-slot bracket. Scanning microscope images revealed that the surface of TiMo was much rougher with abundant scratches visible than that of the NiTi and TiNb wires.

The amount of dynamic friction in dry state at room temperature was measured by Kopsahilis [16] in 660 in vitro tests with 132 different wire-bracket combinations. The loss of applied force due to friction of Gummetal was comparable to well-known archwires as CoCr and SS. No influence of the slot size on friction using different dimensions of Gummetal was found in nine out of twelve results.

### 3.5. Torque Moment

The torque moment provided by Gummetal wire was measured and compared with NiTi and TiMo archwires by Kuroda et al. [17]. Two sizes of TiNb, NiTi, and TiMb and 0.022^″^-slot SS brackets were ligated with elastic modules and ligature wires. The torque moment delivered by the bracket-wire combination was measured by means of a torque gauge at the temperature of 37°C and 50% humidity. The study revealed an increase of the torque moment with increasing torque applied and wire size. The torque moment with elastic ligatures was significantly smaller than that with wire ligatures. With more than 20° torque applied, the torque moment of NiTi and TiMo wires was larger than that of Gummetal wire.

### 3.6. Distribution of Stress and Strain

The evaluation of tension distribution in two different orthodontic mechanical approaches to treat anterior open bite was the aim of the study of Meros et al. [18]. In the in vitro experimental study, the mandibular anterior teeth underwent orthodontic forces provided by Blue Elgiloy 0.016^″^ × 0.022^″^ in multiloop edgewise archwire (MEAW) and Gummetal 0.018^″^ × 0.022^″^ in Gummetal edgewise archwire (GEAW) techniques with and without anterior elastic bands placed between upper lateral incisors and lower canines. The GEAW technique with intermaxillary elastics generated the lowest mean tension values significantly different (*P* < 0.05) from the other groups. GEAW provided lower and more favorable tension levels than MEAW technique with Blue Elgiloy.

The distribution of stresses and deformations in the wire, the bracket, and the dentoalveolar unit with and without class III intermaxillary elastics applied, using Blue Elgiloy archwire with multiloops and Gummetal archwire by finite element analysis, was compared by Jácome et al. [19]. The distributed tension maintained its maximum values at the level of the crest, decreasing towards the mandibular symphysis and towards distal parts of the mandible; trend was shown when using both archwires. The stress and strain distributions for Gummetal and Blue Elgiloy wires were consistent with the distal “en bloc” movement in the teeth and cortical bone. The Blue Elgiloy with multiloops showed higher stress values compared to the Gummetal, when no elastic load was used.

The distribution of stress and strain in the dentoalveolar unit of lower left second molar with 20° inclination with alveolar bone, the wire and the tube with Gummetal and Nitinol by finite element analysis was compared by Pacheco et al. [20]. Gummetal archwire generated less effort (214.28 MPa) than Nitinol (219.93 MPa) and presented slightly smaller (0.007 mm) deformation. The molar, alveolar bone, and molar tube expressed greater stress and strain when using the Nitinol archwire compared to the Gummetal. In conclusion, under the same mechanical conditions, Gummetal showed less effort and deformation than Nitinol.

The comparison of Gummetal to conventional leveling-archwires for the “en bloc” uprighting of mesially inclined premolars and first molar was performed by Bertl et al. [21]. The clinical situation was simulated in a 2D measuring apparatus. Gummetal 0.018^″^ × 0.022^″^ and TMA 0.016^″^ × 0.022^″^ archwires produced similar and highest uprighting moments at the second premolar and highest vertical forces at the first premolar and first molar brackets. Highly significant differences between moments of these two types of archwires and other tested alloys were found. In contrast to other wires, Gummetal, TMA, and Blue Elgiloy multiloop performed the same at room and body temperature.

### 3.7. Calorimetric and Thermomechanical Properties

Gummetal, TMA, Copper NiTi (CuNiTi), Thermalloy Plus, Nitinol SE, and NiTi wires were subjected to a dynamic mechanical analysis and differential scanning calorimetry by Laino et al. [22]. A model was designed to predict the elastic modulus of superelastic wires. Gummetal and TMA presented a flexural elastic modulus almost constant with the temperature. On the contrary, the elastic modulus of the Thermalloy Plus, NiTi, Nitinol, and CuNiti were temperature dependent. It was stated that Gummetal wire behaved as an elastic wire with a very low Young's Modulus (40 GPa+/-3 GPa) which was about half of that related to TMA (105.0+/-8.5 GPa).

## 4. Discussion

*β*-Ti alloys are important class of alloys that have found use in demanding applications such as aircraft structures, engines, orthopedic, and orthodontic implants [23]. Their high strength, great biocompatibility, excellent corrosion resistance, and ease of fabrication provide important advantages compared to other high performance alloys [23]. It was stated by Suzuki et al. [13] that mechanical properties vary markedly among *β*-Ti wires from different manufacturers. Thus, it seems that understanding their specific properties is essential for proper clinical application [13].

Gummetal unveils unique mechanical properties and combines them with typical for *β*-Ti alloys lack of toxicity due to nickel and chromium-free chemical composition [6]. Clinicians should be aware of possible adverse reactions arising from the intraoral use of orthodontic materials due to corrosion, galvanic corrosion, and release of ions from different alloys [24]. TiNb wires could substitute CoCr, SS, and NiTi archwires in particular stages of orthodontic treatment especially, but not only, in susceptible groups of patients [25–27].

Nordstrom et al. [10] claimed that further studies are necessary to evaluate the usefulness of Gummetal with different wire sizes and in various clinical situations in order to prove its advantages over other wires. They also suggested that Gummetal wire could be used in patients when bends could be useful from the beginning of orthodontic treatment.

According to Grauberger [12], advertised “superelasticity” of Gummetal should be discussed and examined in the future.

Orthodontists should be aware of the FFs in bracket-wire combinations to achieve efficient tooth movement [12]. Kopsahilis [16] mentioned that additional in vivo tests with oral mouth conditions might have significant influence on FF rankings of orthodontic wires.

Gentle and continuous load is desired for optimal tooth movement [28]. Gummetal orthodontic archwire could be useful for the initial stage of orthodontic treatment but might be convenient in the final stage as well [22].

The search for an ideal orthodontic wire definitely has not come to an end yet [29]. There are many different methods and tests, including more 3-dimensional finite element model analyses, low-level laser therapies, and biochemical or spectroscopic analytical methods which could be performed to compare the characteristics of novel orthodontic wires with the older existing ones [30–34]. Surprisingly, for the authors of this review, from 2003 when there were first publications about the great potential of Gummetal alloy up to date, to the best knowledge of authors, only one double-blind RCT exists in the literature available to compare its usefulness as orthodontic archwire with dissimilar wire.

## 5. Conclusions

- Gummetal archwire could be useful in the initial stage of orthodontic treatment alternatively to NiTi wires. However, it shows some plastic deformation, which questions its superelasticity
- It is assumed that Gummetal alloy could be used in patients suffering from nickel allergy; all the atomic elements of the alloy are nontoxic and biocompatible
- Gummetal wire exhibits low bending strength, low fatigue limit, and high resilience. However, these properties do not affect the numbers of cycles to fracture (similar risk to wire fracture)
- Loss of applied force due to friction of Gummetal wire is comparable to SS and CoCr wires
- TiNb wires might demonstrate appropriate torque moment and low tension values when they are used combined with edgewise appliances (GEAW technique)
- Gummetal archwire has a very low Young's Modulus constant with the temperature with high tensile strength, which provides lower force than Nitinol and TMA but higher than Supercable wire

## Figures and Tables

**Table 1:** In vivo study.

Authors, year (language)	Study group	Outcome measured	Comparison	Main findings
Nordstrom et al. [10], 2018 (English)	28 patients; age: 12-20	Crowding reduction during initial orthodontic alignment in adolescents over time of 2 months	Fourteen 0.016^″^ NiTi archwires and fourteen 0.016^″^ Gummetal (experimental)	Both wires reduced Little's Irregularity Index (no significant difference between the wires tested), statistically insignificant increase in the transverse dimensions

**Table 2:** In vitro studies.

No	Authors, year (lang.)	Wire type	Size of the wire	Outcome measured	Applied test	Main findings
	Zhang et al. [11], 2019 (English)	Gummetal Ti TC4	0.3 mm	Microstructural and mechanical properties of cold-drawn and annealed TNTZO wires	Optical Microscope and Transmission Electron Microscope (TEM, JEM 2100) used to characterize microstructure. Nanoindentation (UNHT) performed to test elastic modulus and creep behavior	Marble-like cross-section microstructure of cold-drawn TNTZO wire. The tensile strength of cold-drawn TNTZO (1000 MPa) much higher than annealed TNTZO (680 MPa) Similar elastic modulus: cold-drawn 69 GPa, annealed 65 GPa. TNTZO exhibited higher creep resistance and lower stress exponent than Ti and TC4 wires
	Grauberger [12], 2018 (German)	Gummetal Supercable TMA Nitinol Discovery SS brackets 0.018″	0.014^″^ (Gummetal) 0.016^″^ (Supercable) 0,016^″^ (TMA) 0.014 (Nitinol)	Initial force systems investigation of Gummetal and conventional wires	Initial 3D force systems were measured with 3D force moment sensor and RX 60 robot	Amount of force: Supercable < Gummetal < NiTinol < TMA 60% of Gummetal wires showed plastic deformation The forces of NiTi, Gummetal and TMA exceeded the recommended values for leveling
	Suzuki et al. [13], 2015 (Japanese)	Beta-titanium (TiMo), CBA (TiMo), Beta III (TiMo), TitanMoly (TiMo), TMA (TiMo), LOW FRICTION TMA (TiMo), BT3 (TiMo), BENDALOY (TiMo), BETA TITANIUM (TiMo), *β* III (TiMo), Gummetal (Ti-Nb-Ta-Zr-O), TIMOLIUM (Ti-A-V), SS, NEO SENTALLOY F160 (NiTi)	0.016^″^ × 0.022^″^	Bending properties: stiffness, active deflection range, load at 3 mm displacement, plastic deformation	3-point bending test	Gummetal presented: lowest stiffness (below 3 N/mm) second highest active deflection range after NiTi second lowest load at 3 mm deflection (N) after NiTi apparent plastic deformation (only NiTi provided no plastic deformation)
	Murakami et al. [14], 2015 (English)	Gummetal Resolve TMA	0.017^″^ × 0.022^″^ (Gummetal) 0.016^″^ × 0.022^″^ (Resolve) 0.016^″^ × 0.022^″^ (TMA)	Fatigue evaluation, high-cycle fatigue test	Static 3 point bending test with 3 point bending mode SEM observation of fractured wires, micro X-ray diffraction of postfatigue crystal structures	Gummetal exhibited the lowest elastic modulus (44.54 MPa), bending strength (1.241 MPa), fatigue limit (304 MPa) and the highest resilience (0.00086 J).
	Takada et al. [15], 2018 (English)	TiNi, TiNb, TiMo, SS brackets 0.018^″^ and 0.022^″^, elastic modules	0.016^″^ 0.016^″^ × 0.022^″^ 0.017^″^ × 0.025^″^ (0.018^″^-slot-bracket 0.018^″^ 0.017^″^ × 0.025^″^ 0.019^″^ × 0.025^″^ (0.022^″^-slot-bracket	Torque moment delivered by bracket-wire combinations	Dynamic FF at three bracket-wire angles (0°, 5°, 10°) with InStron 5567 loading apparatus	TiNb had almost the same dynamic FFs as the NiTi in 0.018″ bracket. TiMo presented significantly higher (*P* < 0.05) values. FFs were 1.5-2 times lower in 0.022″ bracket regardless of alloy wire type. SEM images showed that the surface of TiMo was much rougher with scratches visible comparing to the other wires
	Kopsahilis [16], 2018 (German)	Gummetal SS Elgiloy NiTi TMA in various brackets: Clarity, Discovery, Inspire Ice, Micro Sprint brackets 0.018^″^ and 0.022^″^, steel wire ligation	0.014^″^ 0.016^″^ 0.016^″^ × 0.022^″^ 0.019^″^ × 0.025^″^	Dynamic friction in the binding modus, dry state, room temperature	Robotic measurement system (RMS) test	Loss of applied force due to friction in Gummetal was comparable to SS and Elgiloy. Friction of Gummetal in Micro Sprint brackets was outstandingly low. Round wires provided lower friction (except for ceramic bracket Inspire Ice) Micro Sprint—low friction
	Kuroda et al. [17], 2014 (English)	TiNb NiTi TiMo, 0.022^″^ SS brackets, elastic modules or ligature wires	0.017^″^ × 0.025^″^ 0.019^″^ × 0.025^″^	Torque moment delivered by bracket-wire combinations	Measuring apparatus consisted of torque transducer connected to a torque gauge	TiNb presented unique torque characteristics - effective torque was smaller than those of TiMo and NiTi when applied torque was larger than 20°
	Meros et al. [18], 2019 (English)	Gummetal Blue Elgiloy Brackets 0018^″^ × 0.025^″^	0.018^″^ × 0.022^″^ (Gummetal) 0.016^″^ × 0.022^″^ (Blue Elgiloy)	Tension distribution in the anterior region of mandible in MEAW and GEAW technique with and without intermaxillary elastics	Tension distribution assessed on photoelastic models simulating lower arch, measured by means of reflection polariscope	Gummetal with elastics generated the lowest mean tension values significantly different (*P* < 0.05) from the other groups MEAW with elastics and GEAW without elastic yielded tension values statistically similar (*P* > 0.05)
	Jácome et al. [19], 2016 (Spanish)	Blue Elgiloy (with multiloops) Gummetal Brackets SS 0.018^″^	Blue Elgiloy 0.016^″^ × 0.022^″^ Gummetal 0.018^″^ × 0.022^″^	Distribution of stress and strain with and without intermaxillary elastics	Mandibular dentoalveolar unit generated by rapid programming and subjected to 300 g force applied by class III elastics (in specialized software)	Gummetal presented lower values of the strains than Blue Elgiloy. The use of elastics did not affect the distribution of stress and strain regardless tested alloy
	Pacheco et al. [20], 2014 (Spanish)	Gummetal Nitinol Buccal tube 0.018^″^ × 0.025^″^	0.018^″^ × 0.022^″^	Distribution of stress and strain of the wires	Simulation of Newton 0.9807 mechanical load applied by mechanical stress in the pipe arch and distributed along the lower left quadrant on simulated observation unit	Gummetal showed less stress and deformation than NiTi under the same mechanical conditions
	Bertl et al. [21], 2013 (German)	Gummetal TMA Blue Elgiloy (with multiloops) Damon CuNiTi Sentalloy Green Sentalloy Black Sentalloy White Standard Edgewise 0.018^″^ brackets	0.018^″^ × 0.022^″^ (Gummetal) 0.016^″^ × 0.022^″^ (Blue Elgiloy) Damon CuNiTi (0.014^″^ × 0.025^″^ and 0.016^″^ × 0.025^″^) Sentalloy Green (0.016^″^ × 0.022^″^) Sentalloy Black and White (0.017^″^ × 0.025^″^)	Uprighting moments produced with different archwires	2D measuring machine with three independent force tranducers for displaying horizontal (*x*-axis) and vertical (*y*-axis) forces, as well as moments around *z*-axis	No significant differences between Gummetal and TMA in producing uprighting moments were observed
	Laino et al. [22], 2012 (English)	Gummetal TMA 35° Copper NiTi Thermalloy Plus Nitinol SE NiTi	0.016^″^ (Gummetal) 0.016^″^ × 0.022^″^ (other alloys)	Mechanical and calorimetric properties of the wires	3-point bending test, dynamic mechanical analysis (DMA) and differential scanning calorimetry (DSC)	Gummetal and TMA presented flexural elastic modulus constant with temperature. TMA elastic modulus (105 GPa) was approximately twice higher than Gummetal's elastic modulus (40 GPa)

## Data Availability

DOI links are available where applicable in the references. The rest of positions in the reference list are books, articles without DOI number (Japanese, German, Spanish), and PhD dissertations available from the corresponding author on reasonable request.

## Abbreviations

*β*-Ti: *β*-Titanium
CoCr: Cobalt-chromium
CuNiTi: Copper nickel-titanium
FF, FFs: Frictional force(s)
GEAW: Gummetal edgewise archwire
MEAW: Multiloop edgewise archwire
NiTi: Nickel-titanium
RCT: Randomized clinical trial
RoB: Risk of bias
SS: Stainless steel
Ti: Titanium
TiMo: Titanium-molybdenum
TiNb: Titanium-niobium, Gummetal
TNTZO: Titanium-niobium-tantalum-zirconium-O, Gummetal.
